# Effects of Ranibizumab combined with laser photocoagulation on macular volume and best corrected visual acuity in patients with macular edema secondary to ischemic retinal vein occlusion

**DOI:** 10.12669/pjms.39.2.7040

**Published:** 2023

**Authors:** Jing Liang, Ting Xiong, Huiqiong Chen, Ran Tao, Liping Cao

**Affiliations:** 1 Jing Liang, Department of Ophthalmology, Shenzhen Hospital, Southern Medical University, Shenzhen, Guangdong 518000, P.R. China; 2 Ting Xiong, Department of Ophthalmology, Huazhong University of Science and Technology Union Shenzhen Hospital, Shenzhen, Guangdong 518000, P.R. China; 3 Huiqiong Chen, Department of Ophthalmology, Shenzhen Hospital, Southern Medical University, Shenzhen, Guangdong 518000, P.R. China; 4 Ran Tao, Department of Ophthalmology, Shenzhen Hospital, Southern Medical University, Shenzhen, Guangdong 518000, P.R. China; 5 Liping Cao, Department of Ophthalmology, Shenzhen Hospital, Southern Medical University, Shenzhen, Guangdong 518000, P.R. China

**Keywords:** Retinal vein occlusion, Macular edema, Ranibizumab, Laser photocoagulation, Macular volume

## Abstract

**Objective:**

To investigate the effects of ranibizumab combined with laser photocoagulation on macular volume and best corrected visual acuity (BCVA) in patients with macular edema secondary to ischemic retinal vein occlusion.

**Methods:**

The clinical data of 90 patients (90 eyes) with macular edema secondary to ischemic retinal vein occlusion treated in our hospital from June 2018 to December 2021 were retrospectively analyzed. Patients were divided into Groups-A, B, and C according to the type of treatment they received. The Group-A was treated with laser photocoagulation, the Group-B was intravitreally injected with ranibizumab, and the Group-C underwent ranibizumab combined with laser photocoagulation. The efficacy, intraocular pressure, BCVA, central macular thickness (CMT) and adverse reactions were compared among the three groups.

**Results:**

The total efficacy of the Group-C was higher than that of the Group-A and B, with statistically significant differences (*P*< 0.05). Three months after treatment, BCVA was higher while CMT was reduced in the Group-C than those in the Group-A and B (*P* < 0.05). Six months after treatment, BCVA was higher while intraocular pressure was lower and CMT was thinner in the group C compared with the Group-A and B (*P*< 0.05).

**Conclusions:**

Ranibizumab combined with laser photocoagulation in the treatment of macular edema secondary to ischemic retinal vein occlusion presents significant efficacy, and can effectively reduce macular volume, improve visual acuity and promote the recovery of retinal function.

## INTRODUCTION

Retinal vein occlusion (RVO) is a common vascular disease of the ocular fundus in the clinic, including branch retinal vein occlusion (RVO) and central retinal vein occlusion (CRVO), the latter of which has a relatively low incidence but a relatively poor visual prognosis and a higher likelihood of developing neovascular glaucoma.^1^,^2^ According to an epidemiological investigation, secondary macular edema is the main cause of vision loss and even blindness in patients with ischemic RVO, which has a serious impact on the daily life of patients.^3^,^4^ Therefore, timely and effective treatment is conducive to improving the prognosis of patients. In the absence of anti-vascular endothelial growth factor (VEGF) drugs, macular grid laser therapy was the standard treatment of RVO.^5^

However, a long-term study has proved that receiving retinal laser treatment will cause damage to the macular region at varying degrees.^6^ Intravitreal injection of ranibizumab can repair capillary endothelial cells by inhibiting the expression of VEGF, thereby alleviating the damage to the blood-retinal barrier.^7^ Nevertheless, it has been demonstrated that the half-life period of ranibizumab is short, and multiple repeated injections are required for clinical treatment, which will increase the risk of intraocular infection.^8^ On this basis, this study aimed to investigate the efficacy of ranibizumab combined with laser photocoagulation in patients with macular edema secondary to ischemic RVO.

## METHODS

The clinical data of 90 patients (90 eyes) with macular edema secondary to ischemic RVO treated in our hospital from June 2018 to December 2021 were retrospectively analyzed. This study has been approved by the medical ethics committee of Ethical Approval (No.:2021030; date: May 12, 2021): Huazhong University of Science and Technology Union Shenzhen Hospital and written informed consent was obtained from all participants.

### Inclusion criteria:

- Patients of RVO confirmed by fundus examination, optical coherence tomography (OCT) and fundus fluorescein angiography (FFA). Moreover, FFA showed a large number of capillaries without perfusion area, the nonperfusion area of retina is larger than five optic discs, which was clearly ischemic RVO.
- Best corrected visual acuity (BCVA) ≥ 0.1 examined by the international standard visual acuity chart.
- Unilateral involvement;
- Complete clinical data.

### Exclusion criteria:

- A previous history of intravitreous injection and retinal photocoagulation.
- Obvious vitreous or lens opacity.
- Complicated cardio renal insufficiency and diabetes mellitus.
- Complicated retinal diseases affecting visual acuity (VA).
- A history of intraocular surgery and trauma.

Patients were divided into groups A, B, and C according to the type of treatment they received, with 30 patients in each group. The Group-A was treated with laser photocoagulation, the Group-B was intravitreally injected with ranibizumab, and the Group-C underwent ranibizumab combined with laser photocoagulation. The general data of the three groups showed no significant differences (*P* >0.05), as seen in Table-I.

**Table-I:** Comparison of general data.

Groups	Gender (male/female)	Age (year)	Course of disease (month)	Volume of macular edema (mm^2^)	Diastolic pressure (mmHg)	Systolic pressure (mmHg)	Location	

							Left eye	Right eye
Group-A (30)	12/18	50.65 ± 6.13	1.71 ± 0.45	0.59 ± 0.17	84.81 ± 12.44	135.8 ± 14.45	15 (50)	15 (50)
Group-B (30)	11/19	51.83 ± 7.15	1.66 ± 0.46	0.59 ± 0.19	83.75 ± 12.56	136.45 ± 15.05	14 (46.67)	16 (53.33)
Group-C (30)	17/13	51.8 ± 7.14	1.62 ± 0.36	0.60 ± 0.23	84.19 ± 12.55	135.95 ± 14.77	15 (50)	15 (50)
*F*/*χ^2^*	0.104	0.437	0.505	0.038	0.081	0.024	0.011	
*P*	0.747	0.647	0.605	0.963	0.922	0.976	0.916	

Before treatment, the three groups were all treated with compound tropicamide eye drops (Shenyang Xingqi Pharmaceutical Co., Ltd., Guoyao Zhunzi H20055546) for mydriasis, and 0.5% proparacaine hydrochloride eye drops (s.a.ALCON-COUVREUR n.v) for topical anesthesia. The Group-A was treated with laser photocoagulation: “C-shaped” grid photocoagulation was performed in the macular region using an ophthalmic semiconductor laser photocoagulator to be Multiwavelength laser photocoagulator (MC-500, Nidek Medical Device Trading Co., Ltd., Shanghai), with the yellow wavelength of 568 nm, exposure time of 0.10~0.15 s, the spot diameter of 100 µm and energy of 100~230 mW. Ensuring that the macular bundles of the optic disc were spared > 500 µm away from the macular center, light spots with power I~II were outputted, with the spacing set to one spot diameter. If the scope of the non-perfusion area is large, the laser can be divided into multiple treatments, one week apart. All laser treatments were completed within one month.

The Group-B was intravitreally injected with ranibizumab: Routine disinfection and towel paving were conducted in the supine position. The needle was inserted into the flat part of the ciliary body 3.5mm behind the corneoscleral margin of the inferior temporal angle, and 0.5mg ranibizumab (Novartis Pharma Stein AG, Switzerland, Registration Certificate No. S20170003) was injected into the vitreum vertical to the scleral surface. Once a month for three consecutive times. The interval between every two treatments should be one month. If the patient cannot see the doctor in time, the delay of vitreous cavity injection should not exceed one week. The Group-C underwent combined treatment: Laser photocoagulation was performed one week after the first intravitreal injection of ranibizumab. The treatment method and course of intravitreal injection of ranibizumab in Group-C were the same as those in Group-B. Iop was measured 30 minutes after treatment in all patients, and was remeasured one hour later in patients with intraocular pressure exceeding 21mmHg. For patients with normal IOP retested one hour later, it was defined as transient ocular hypertension. The three groups were all reexamined one, three and six months after treatment. Follow-up measures included intraocular pressure, BCVA and CMT. Six months after the end of treatment, the efficacy of each group was counted by outpatient reexamination:^9^

### Remarkably effective:

Retinal hemorrhage was well absorbed, and no neovascularization was detected in the retina or non-perfusion area by FFA, without fluorescein leakage;

### Effective:

FFA detected a nonperfusion area in the retina, but the nonperfusion area was smaller than three optic discs;

### Ineffective:

FFA detected non-perfusion areas of the retina with an area larger than three optic discs. Effective rate = (remarkably effective cases + effective cases)/Total number of people in the group. The intraocular pressure, BCVA, central macular thickness (CMT) was compared among the three groups before, three and six months after treatment.

The adverse reactions were compared among each group, including cataract, transient ocular hypertension, transient vitreous opacity, subconjunctival hemorrhage and retinal detachment.

### Statistical Analysis:

The data were analyzed using SPSS 22.0. The enumeration data were expressed as (*n*, %), and analyzed with the *χ^2^* test and rank-sum test. The measurement data following the normal distribution were expressed as (), and inter-group and intra-group comparisons were carried out using the independent sample t-test and paired samples t-test, respectively. *P*<0.05 was considered statistically significant.

## RESULTS

The total efficacy of the group C was higher than that of the Group-A and B (93.33% vs. 70.00%, 73.33%), with statistically significant differences (*P* < 0.05), as seen in Table-II. The intra-group comparison showed that intraocular pressure and CMT three and six months after treatment all reduced significantly, and BCVA increased significantly six months after treatment in the three groups than those before treatment (*P<*0.05). Three and six months after treatment, BCVA was higher while CMT was thinner in the Group-C than those in the Group-A and B (*P*<0.05), and six months after treatment, intraocular pressure was lower than the Group-A and B (*P*<0.05). Three and six months after treatment, CMT in the Group-B was thicker than that in the Group-A (*P*<0.05) (Table-III). No statistically significant differences were found in adverse reactions among the three groups (*P >* 0.05), as shown in Table-IV.

**Table-II:** Comparison of efficacy among the three groups (*n*, %).

Groups	Remarkably effective	Effective	Ineffective	Total effective rate
Group-A (30)	8 (26.67)	13 (43.33)	9 (30.00)	21 (70.00)
Group-B (30)	8 (26.67)	14 (46.67)	8 (26.67)	22 (73.33)
Group-C (30)	12 (40.00)	16 (53.33)	2 (6.67)^*#^	28 (93.33)^*#^
*Z/χ^2^*	4.073			4.153
*P*	0.044			0.042

***Notes:*** Compared with the group A, P^*^ < 0.05; compared with the group B, *P*^#^ < 0.05.

**Table-III:** Comparison of intraocular pressure, BCVA and CMT among the three groups

Index	Groups	Before treatment	3 months after treatment	6 months after treatment	F	P
Intraocular pressure (mmHg)	Group-A (30)	15.35 ± 2.76	14.04 ± 2.17*^$^*	13.10 ± 1.92*^$^*	*F*_time point_ = 146.099 *F*_inter-group_ = 2.144 *F*_cross_ = 5.661	*P*_time point_ < 0.001 *P*_inter-group_ = 0.121 *P*_cross_ = 0.004
	Group-B (30)	15.33 ± 2.63	14.38 ± 2.14*^$^*	13.80 ± 2.01*^$^*		
	Group-C (30)	15.22 ± 2.61	13.64 ± 2.16*^$^*	12.13 ± 1.69^*#*$*^		
	*F*	0.031	1.327	8.972		
	*P*	0.969	0.269	< 0.001		
BCVA	Group-A (30)	0.48 ± 0.08	0.48 ± 0.07	0.58 ± 0.05	*F*_time point_ = 521.474 *F*_inter-group_ = 12.787 *F*_cross_ = 7.237	*P*_time point_ < 0.001 *P*_inter-group_ < 0.001 *P*_cross =_ 0.001
	Group-B (30)	0.45 ± 0.08	0.50 ± 0.07	0.57 ± 0.05*^$^*		
	Group-C (30)	0.48 ± 0.07	0.57 ± 0.06^*#^	0.63 ± 0.05^*#*$*^		
	*F*	6.864	22.500	18.600		
	*P*	0.001	< 0.001	< 0.001		
CMT (µm)	Group-A (30)	549.01 ± 92.79	304.37 ± 59.22*^$^*	246.23 ± 30.08*^$^*	*P*_time point_ < 0.001 *P*_inter-group_ < 0.001 *P*_cross_ < 0.001
	Group-B (30)	534.33 ± 93.23	359.21 ± 78.03^*$^	300.26 ± 39.08^*$^	
	Group-C (30)	561.49 ± 94.3	209.02 ± 30.55^*#*$*^	208.21 ± 29.38^*#*$*^	
	*F*	0.953	74.059	87.658	
	*P*	0.388	< 0.001	< 0.001	

***Notes:*** Compared with the group A at the same time point, P^*^ < 0.05; compared with the group B at the same time point, *P*^#^ < 0.05; compared with before treatment, *P^$^* < 0.05.

**Table-IV:** Comparison of adverse reactions among the three groups

Groups	Cataract	Transient ocular hypertension	Transient vitreous opacity	Subconjunctival hemorrhage	Retinal detachment	Total incidence
Group-A (30)	0 (0.00)	1 (3.33)	1 (3.33)	2 (6.67)	0 (0.00)	4 (13.33)
Group-B (30)	1 (3.33)	1 (3.33)	1 (3.33)	1 (3.33)	0 (0.00)	4 (13.33)
Group-C (30)	0 (0.00)	1 (3.33)	1 (3.33)	1 (3.33)	0 (0.00)	3 (10.00)
*χ^2^*	-	-	-	-	-	0.026
*P*	-	-	-	-	-	0.872

## DISCUSSION

This study retrospectively analyzed the efficacy of intravitreal ranibizumab combined with laser photocoagulation in patients with RVO secondary macular edema. The results showed that intravitreous injection of ranibizumab combined with laser photocoagulation has a good effect in the treatment of macular edema secondary to RVO. The results of follow-up showed that it had good safety. According to the different degree of ischemia, RVO can be divided into non-deficient blood group and deficient blood group. Compared with non-deficient blood group, deficient blood group is characterized by poor baseline vision, visual field defect, and larger non-perfusion area of retinal capillaries, which greatly harm vision.^10^,^11^ It has been shown that in patients with macular edema secondary to ischemic RVO, the function of the retinal photoreceptor is damaged due to macular cell dysfunction, which can lead to vision loss or even blindness without timely control.^12^

As early as 1984, the RVO research team in the United States, with an average follow-up of 3.1 years, found that grating photocoagulation was effective in the treatment of macular edema secondary to RVO, and recommended that it be made the standard treatment for RVO.^13^ But its ability to improve vision has been controversial. Campochiaro et al. reported that laser did not significantly improve BCVA in RVO patients.^14^ In this study, after receiving laser photocoagulation therapy, IOP and CMT in group A were significantly reduced, and BCVA was significantly increased compared with before treatment. The cause may be that laser photocoagulation can alleviate ischemia and hypoxia in the non-perfusion area of the retina, reduce blood reflux and improve symptoms.^15^

VEGF inhibitors in the treatment of macular edema have become a new hot spot in clinical research. Ranibizumab is the second-generation humanized mouse anti-VEGF monoclonal antibody.^16^ In this study, IOP and CMT of group B were significantly reduced and BCVA was significantly increased after intravitreal injection of ranibizumab. It has been shown that intravitreous injection of ranibizumab can avoid the interference of the blood-retinal barrier, block the VEGF-A receptor for a long time, and effectively improve the thickness of macular edema and the degree of RVO. Ranibizumab can inhibit angiogenesis, regulate the blood-retinal barrier, and reduce vascular permeability, thus promoting retinal exudate absorption and alleviating symptoms.^17^ However, the maintenance of its efficacy is short, so it needs repeated injections, which increase the economic burden on patients, and limit its clinical promotion.^18^ The CMT of the Group-B was thicker than that of Group-A three and six months after treatment, which may be related to the regression of the efficacy of ranibizumab.

On this basis, our study creatively proposed an intravitreous injection of ranibizumab combined with laser photocoagulation in the treatment of macular edema secondary to RVO. Their combination makes up for their respective shortcomings, and plays a synergistic role, resulting in a more obvious effect. Three months after treatment, BCVA was higher while CMT was reduced in the Group-C than those in the Group-A and B, and six months after treatment, intraocular pressure was lower than the Group-A and B. These results further suggest that the effect of combined treatment is more advantageous than that of a single treatment. Goel S et al. has reported that ranibizumab combined with laser could improve BCVA and CMT in RVO patients and reduce the number of injection times, which was consistent with the conclusion of this study.^19^ Studies have shown that intravitreal injection of ranibizumab before laser photocoagulation can reduce the thickness of macular edema, while laser photocoagulation can improve the durability and stability of the efficacy of ranibizumab.^20^ The results of our study indicate that combined treatment can effectively improve the retinal function. Which was similar to the conclusions of previous studies.^21^ Moreover, no significant differences were found in adverse reactions among the three groups, suggesting the safety of combined treatment.

The conclusion of this study provided a new clinical reference for the treatment of RVO secondary macular edema. Follow-up studies are needed to further summarize the timing and efficacy of laser therapy.

### Limitations

It includes small sample size, so it still needs to be further verified by a large-sample study.

## CONCLUSION

Ranibizumab combined with laser photocoagulation in the treatment of macular edema secondary to ischemic RVO presents significant efficacy, and can effectively reduce macular volume, improve VA and promote the recovery of retinal function.

### Authors’ Contributions:

**JL and** **TX** carried out the studies, participated in collecting data, and drafted the manuscript, and are responsible and accountable for the accuracy or integrity of the work.

**HC and** **RT** performed the statistical analysis and participated in its design.

**LC** participated in acquisition, analysis, or interpretation of data and draft the manuscript.

All authors read and approved the final manuscript.
